# A new species of *Messor* from Bulgaria and redescription of the gyne of *M. oertzeni* Forel, 1910 (Hymenoptera, Formicidae)

**DOI:** 10.3897/zookeys.1275.181745

**Published:** 2026-03-27

**Authors:** Albena Lapeva-Gjonova, Lech Borowiec

**Affiliations:** 1 Faculty of Biology, Sofia University, 8 Dragan Tsankov str., 1164 Sofia, Bulgaria Myrmecological Laboratory, Department of Biodiversity and Evolutionary Taxonomy, University of Wrocław Wrocław Poland; 2 Myrmecological Laboratory, Department of Biodiversity and Evolutionary Taxonomy, University of Wrocław, Przybyszewskiego 65, 51–148 Wrocław, Poland Faculty of Biology, Sofia University Sofia Bulgaria

**Keywords:** Ants, COI, key, morphology, taxonomy, the Balkans

## Abstract

A new ant species, *Messor odrysarum***sp. nov**., of the *Messor structor* species group, is described based on worker, gyne and male castes collected in Eastern Rhodopes, Bulgaria. The description is supported by the morphology of all castes and COI barcoding. Both the morphological traits and COI sequence data indicate that the new species is closely related to *Messor oertzeni* Forel, 1910, a species restricted to the eastern Balkans and Anatolia. Furthermore, the little-known gyne of *M. oertzeni* was redescribed based on a specimen collected in Bulgaria. The new data increases the number of *Messor* species known from Bulgaria to nine, for which an updated key is provided.

## Introduction

Ants of the genus *Messor* Forel, 1890 have drawn considerable research attention due to their role as grain collectors in arid and semi-arid ecosystems, as well as their intriguing taxonomy and biology, including cryptic alpha diversity and remarkable reproductive strategies such as hybridisation, social hybridogenesis and even xenoparity (Steiner et al. 2011; Romiguier et al. 2017; Steiner et al. 2018; Juvé et al. 2025a). In search of resolving the taxonomic and phylogenetic problems within the genus, caused by the intraspecific variation, polymorphism, and cases of hybridisation, significant progress has recently been made with revisions of certain groups of species (Steiner et al. 2018; Salata et al. 2023b), studies of specific geographical areas (Salata and Borowiec 2019; Barech et al. 2020; Orou et al. 2023; Saar et al. 2023; Salata et al. 2023a; Borowiec and Salata 2025; Lapeva-Gjonova et al. 2025) and results from phylogenomics (Juvé et al. 2025b).

With 134 species within the Palearctic, Afrotropical and Oriental biogeographic regions, the greatest diversity of *Messor* species is currently found in North Africa and the Middle East (Bolton 2025; Juvé et al. 2025a). In the Balkans, the genus reaches its highest species richness in Greece and Bulgaria, with at least 16 and 8 species, respectively, though the taxonomic status of a few species remains unclear, highlighting the need for integrative studies incorporating nuclear genetic data (Lapeva-Gjonova and Borowiec 2022; Borowiec and Salata 2025; Lapeva-Gjonova et al. 2025). The two countries share eight species in common, while the remaining eight occur on the Greek islands. The following eight species of *Messor* ants have been recorded from Bulgaria and continental part of Greece: *M. atanassovii* Atanassov, 1982 (with type locality in Bulgaria), *M. wasmanni* Krausse, 1910, *M. oertzeni* Forel, 1910, *M. ibericus* Santschi, 1931, *M. ponticus* Steiner et al., 2018 (with type locality in Bulgaria), *M. hellenius* Agosti & Collingwood, 1987 (with type locality in Greece), *M. mcarthuri* Steiner et al., 2018 and *M. structor* (Latreille, 1798). The last six of these species belong to the *Messor structor* group, whose detailed characterisation was provided by Steiner et al. (2018).

A recent study of the *Messor* genus, focusing particularly on Bulgarian species, revealed three additional morphotypes through the analysis of morphology and COI barcodes (Lapeva-Gjonova et al. 2025). While further supporting studies are needed for the status of two, here we provide a detailed morphological description accompanied by COI data and biological and diagnostic data for one of them, confirming its status as a new species.

## Material and methods

In this study, 10 continuous morphometric traits were recorded in 35 workers (20 from the largest major and 15 from the smaller ones), 3 gynes and 3 males of *Messor odrysarum* sp. nov. from four collecting sites, as well as in one dealate gyne of *M. oertzeni* from Bulgaria.

Measurements were taken using a Nikon SMZ1270i stereomicroscope at magnifications of × 40 to × 80, with a Nikon DS-Ri2 camera and NIS-Elements D software (v. 5.30). Body size measurements are given in millimetres as mean ± standard deviation, with minimum and maximum values shown in brackets. The measurements follow Borowiec and Salata (2025) and were applied to *Messor* species, with the addition of hind femur length, petiole length, and petiole height. They are listed below in alphabetical order:

**EL** eye length; measured along maximum diameter of eye;

**HFL** hind femur length; measured on dorsal side from trochanter to apex of femur;

**HL** head length; maximum cephalic length in full-face view, measured as a straight line from midpoint of anterior clypeal margin to midpoint of posterior margin. Excavations of posterior head and/or clypeus reduce HL;

**HW** head width; maximum cephalic width in full-face view, measured directly above eyes;

**MW** mesosoma width; maximum mesosoma width in dorsal view; in workers maximum pronotal width;

**PSL** propodeal spine length; measured in lateral view from center of propodeal spiracle to tip of spine, or to propodeal angle in specimens without a distinct spine;

**PTH** petiole height; measured in lateral view from midpoint of basal margin to apex of the node;

**PTL** petiole length; maximum length of petiole in lateral view, from anterior margin of peduncle to posterior margin of node;

**SL** scape length; maximum straight-line length of scape, excluding basal condylar bulb;

**WL** Weber’s length; diagonal length from anterior end of pronotal convexity to posterior margin of propodeal lobe.

The following ratios were used: **HL/HW**; **SL/HW**; **PTH/PTL**; **HFL/WL**.

Abbreviations used: **w**. - worker/s; **g**. – gyne/s; **m**. – male/s.

The degree of pilosity inclination follows the system used by Wilson (1955). Worker body measurements and details of the morphology of the new species were compared with those reported for *M. oertzeni* by Borowiec and Salata (2025) and examined specimens from Bulgaria and Greece. To date, the only known gyne of *M. oertzeni* (https://www.antweb.org/specimen/CASENT0904136) corresponds to the specimen described as *M. oertzeni amasiensis* Emery, 1921 from Amasia, Türkiye (Emery 1921).

A COI sequence of the new species (under provisional name *Messor* sp. 2) was previously published by Lapeva-Gjonova et al. (2025) (BOLD accession BGANT009-23, BIN: BOLD:AES9246). This sequence was used in the same study to calculate pairwise genetic distance under the Kimura 2-parameter model in MEGA v. 12, and phylogenetic reconstruction was performed using both maximum likelihood (ML) and Bayesian inference (BI) analyses (see Lapeva-Gjonova et al. 2025 for details).

Photos of the new species were taken with a Nikon SMZ 1500 stereomicroscope, Nikon D5200 camera, and then aligned and stacked using Helicon Focus v. 8.2.0. Photos of *M. oertzeni* gyne were taken with a Nikon DS-Ri2 camera through a Nikon SMZ 1270i stereomicroscope and then aligned and stacked using Helicon Focus v. 8.3.6.

Examined specimens are housed in the collections of the Biological Faculty, University of Sofia, Bulgaria (BFUS) and Museum of Natural History, University of Wroclaw, Wroclaw, Poland (MNHW).

## Results

### Messor odrysarum

Taxon classificationAnimaliaHymenopteraFormicidae

Lapeva-Gjonova & Borowiec sp. nov.

2E86DCB9-4DE3-5396-B6D2-BADD1EF2A2B0

https://zoobank.org/889C0689-FD15-4BFD-B709-ED6FB3338BF0

Figs 1, 2, 3, 4, 5, 6, 7, 8, 9, 10

#### Material examined.

***Holotype***: Bulgaria • 1 w. (pin), East Rhodopes, Kardzhali district, Sokolyane, 41°45.38'N, 25°25.92'E, alt. 468 m, 15 Sept. 2025, leg. A. Lapeva-Gjonova, BFUS Collection, Catalog Number: BFUS-I-AG003233. ***Paratypes***: BULGARIA • 7 w. (pin): the same locality as holotype, 25 Aug. 2022, leg. A. Lapeva-Gjonova (BFUS: from BFUS-I-AG003201 to BFUS-I-AG003207); • 14 w. (pin): the same locality as holotype, 03 June 2025, leg. A. Lapeva-Gjonova (BFUS: from BFUS-I-AG003208 to BFUS-I-AG003221); • 4 w. (pin): the same locality as holotype, 03 June 2025, leg. A. Lapeva-Gjonova (MNHW); • 1 g. (pin) (BFUS: BFUS-I-AG003229), 3 m. (pin) (BFUS: from BFUS-I-AG003230 to BFUS-I-AG003232), 8 w. (pin) (BFUS: from BFUS-I-AG003221 to BFUS-I-AG003228): the same data as holotype, leg. A. Lapeva-Gjonova; • 1 g., 1 m., 3 w. (pin): the same data as holotype, leg. A. Lapeva-Gjonova (MNHW); • 4 w. (3 EtOH/1 pin): Eastern Rhodopes, Kardzhali district, Oreshari vill., 41°36.59'N, 25°43.04'E, alt. 111 m, 22 Apr. 2014, leg. A. Lapeva-Gjonova (MNHW); • 1 w. (pin): Eastern Rhodopes, Haskovo district, Chernichino vill., 41°35.35'N, 25°50.91'E, 647 m, 23 Apr. 2021, leg. A. Lapeva-Gjonova (MNHW); • 5 w. (pin): same locality as previous, 15. Nov. 2025, leg. A. Lapeva-Gjonova (BFUS: from BFUS-I-AG003196 to BFUS-I-AG003200); • 5 w. (pin): Eastern Rhodopes, Kardzhali district, Maslinovo vill., 41°46.03'N, 25°32.56' E, alt. 362 m, 03 June 2025, leg. A. Lapeva-Gjonova (MNHW).

**Figure 1.**
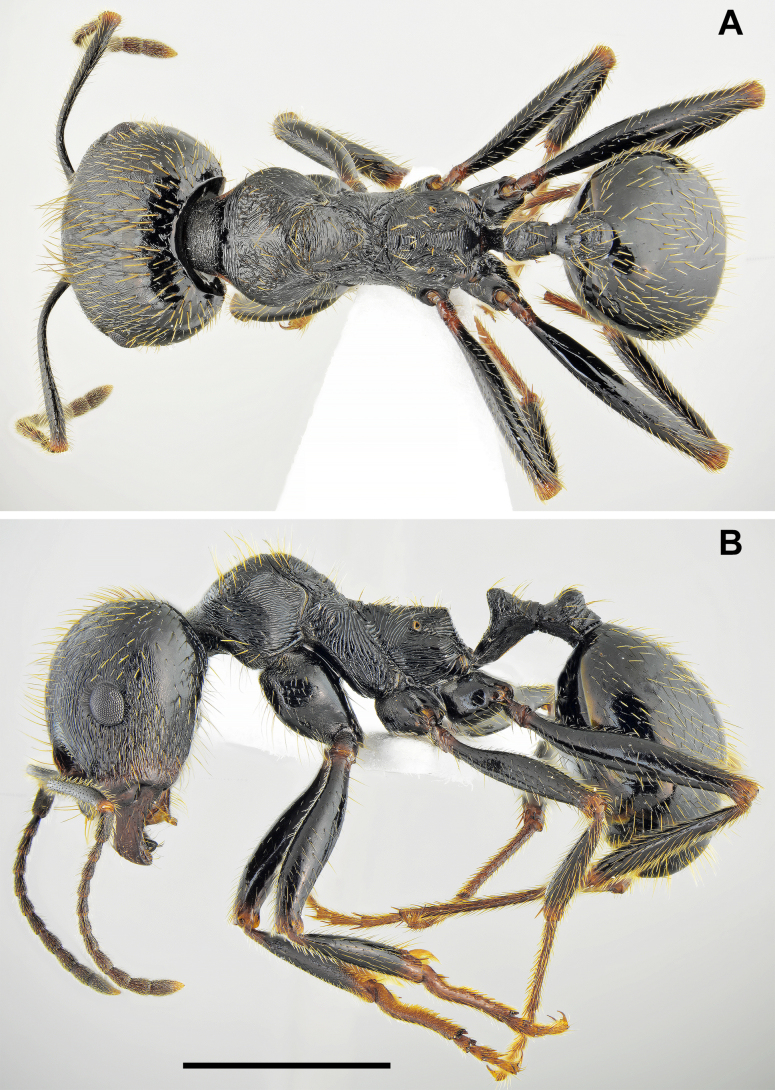
*Messor odrysarum* sp. nov., major worker: **A**. dorsal view; **B**. lateral view (photographed by L. Borowiec). Scale bar: 2 mm.

**Figure 2.**
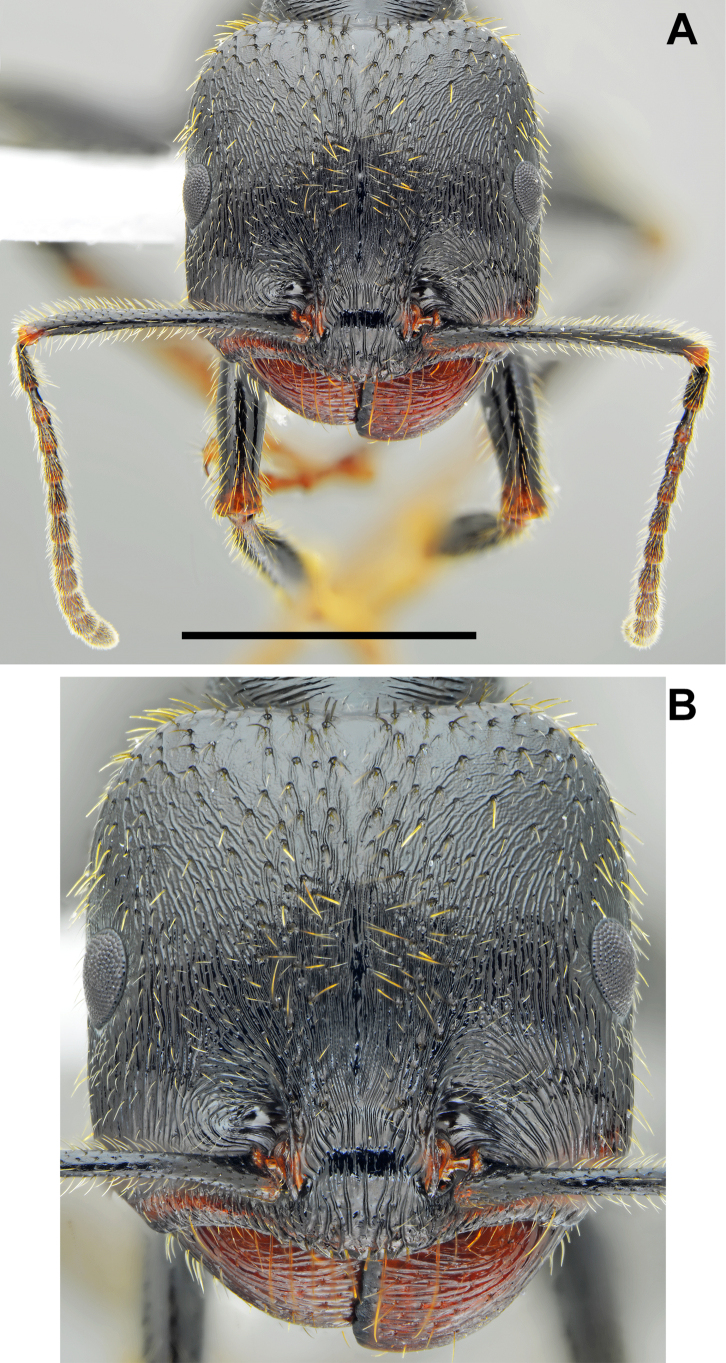
*Messor odrysarum* sp. nov., major worker: **A**. head and antennae; **B**. head sculpture (photographed by L. Borowiec). Scale bar: 2 mm.

**Figure 3.**
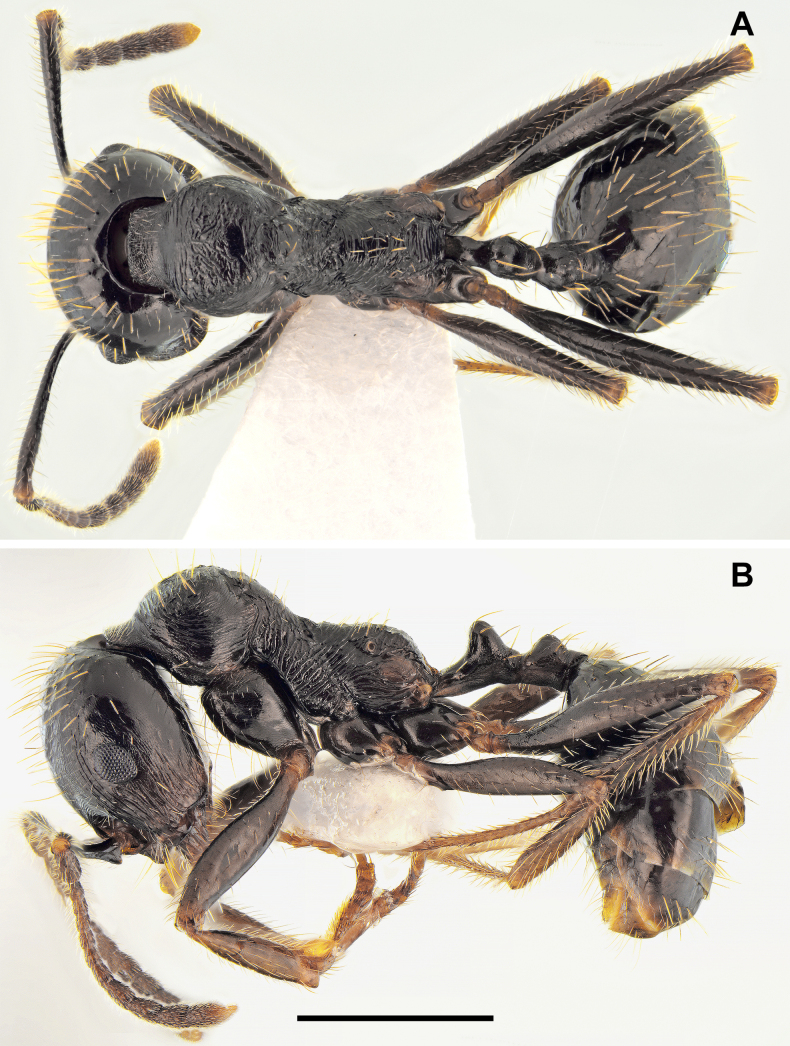
*Messor odrysarum* sp. nov., minor worker: **A**. dorsal view; **B**. lateral view (photographed by L. Borowiec). Scale bar: 1 mm.

**Figure 4.**
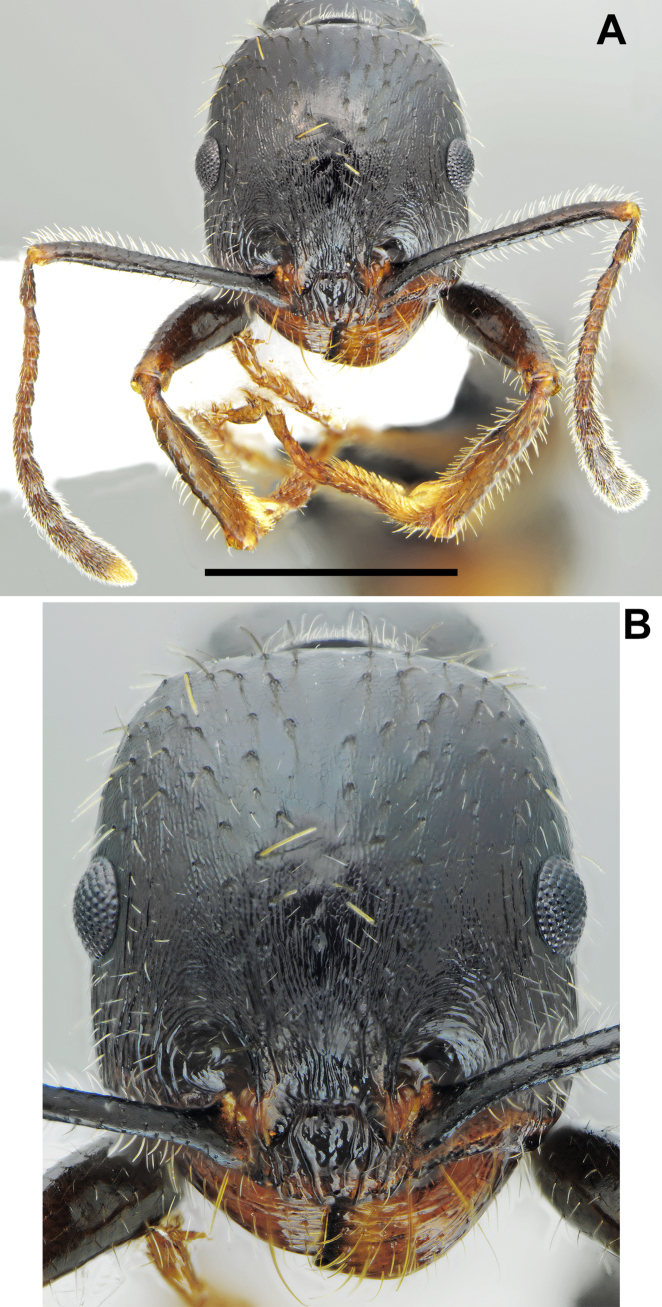
*Messor odrysarum* sp. nov., minor worker: **A**. head and antennae; **B**. head sculpture (photographed by L. Borowiec). Scale bar: 1 mm.

**Figure 5.**
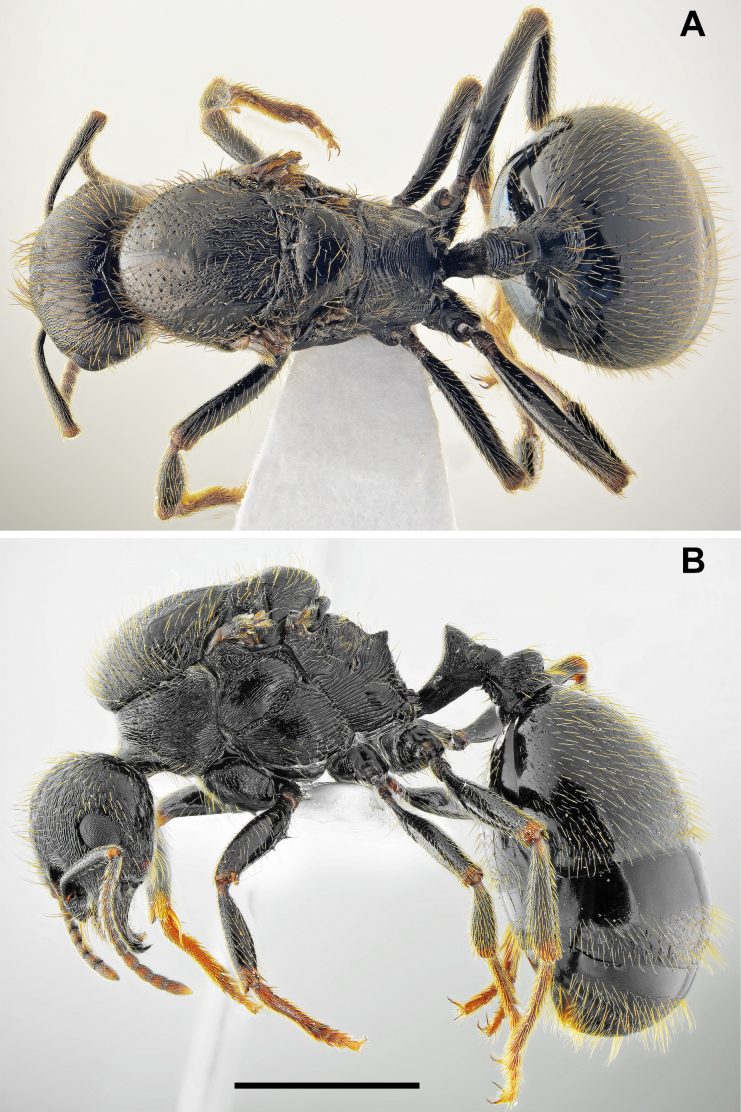
*Messor odrysarum* sp. nov., gyne: **A**. dorsal view; **B**. lateral view (photographed by L. Borowiec). Scale bar: 2 mm.

**Figure 6.**
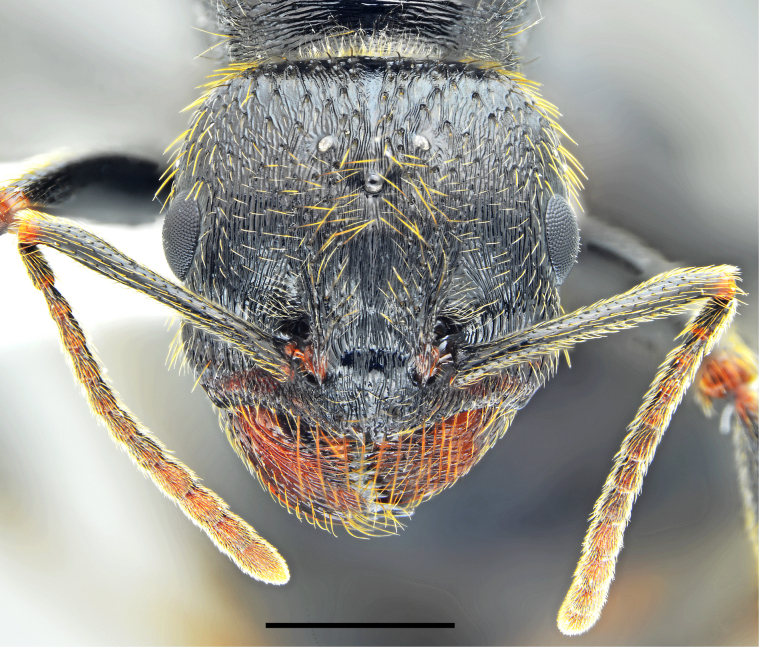
*Messor odrysarum* sp. nov., gyne head (photographed by L. Borowiec). Scale bar: 1 mm.

**Figure 7.**
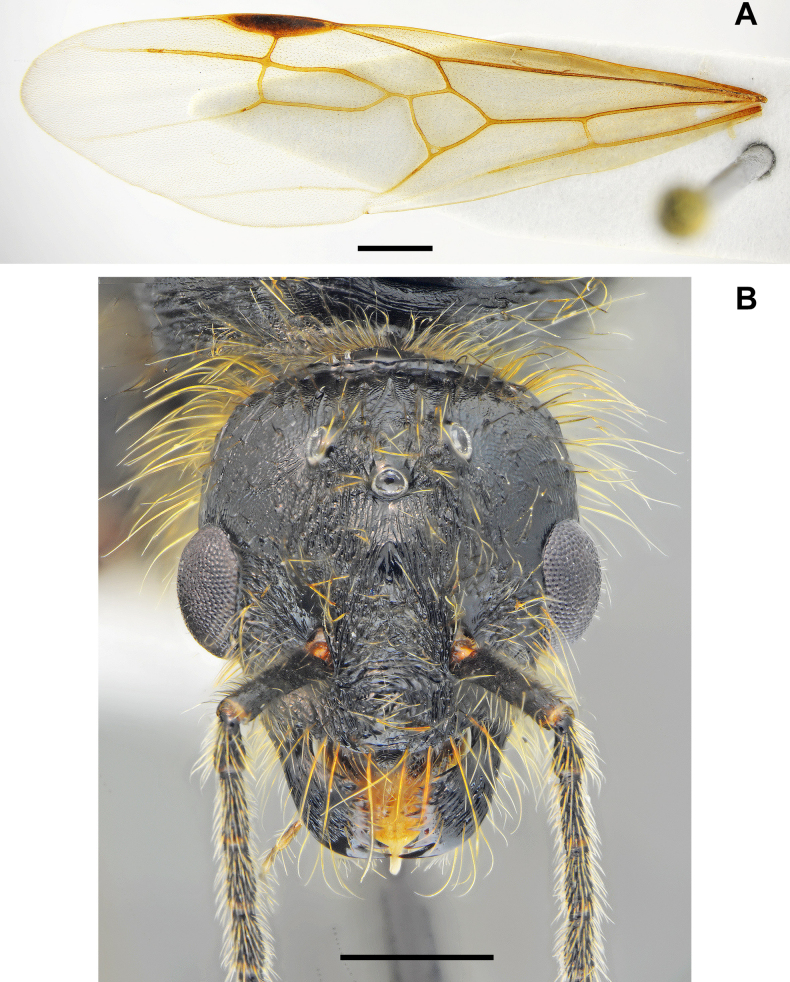
*Messor odrysarum* sp. nov: **A**. gyne forewing; **B**. male head (photographed by L. Borowiec). Scale bars: 1 mm (A); 0.5 mm (B).

**Figure 8.**
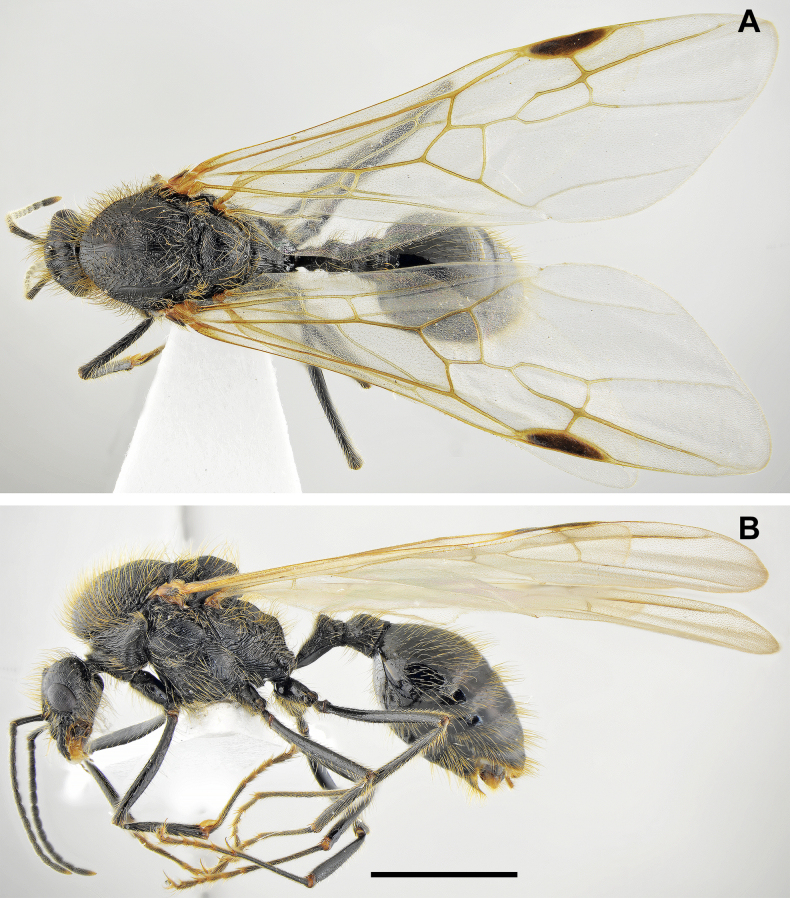
*Messor odrysarum* sp. nov., male: **A**. dorsal view; **B**. lateral view (photographed by L. Borowiec). Scale bar: 2 mm.

**Figure 9.**
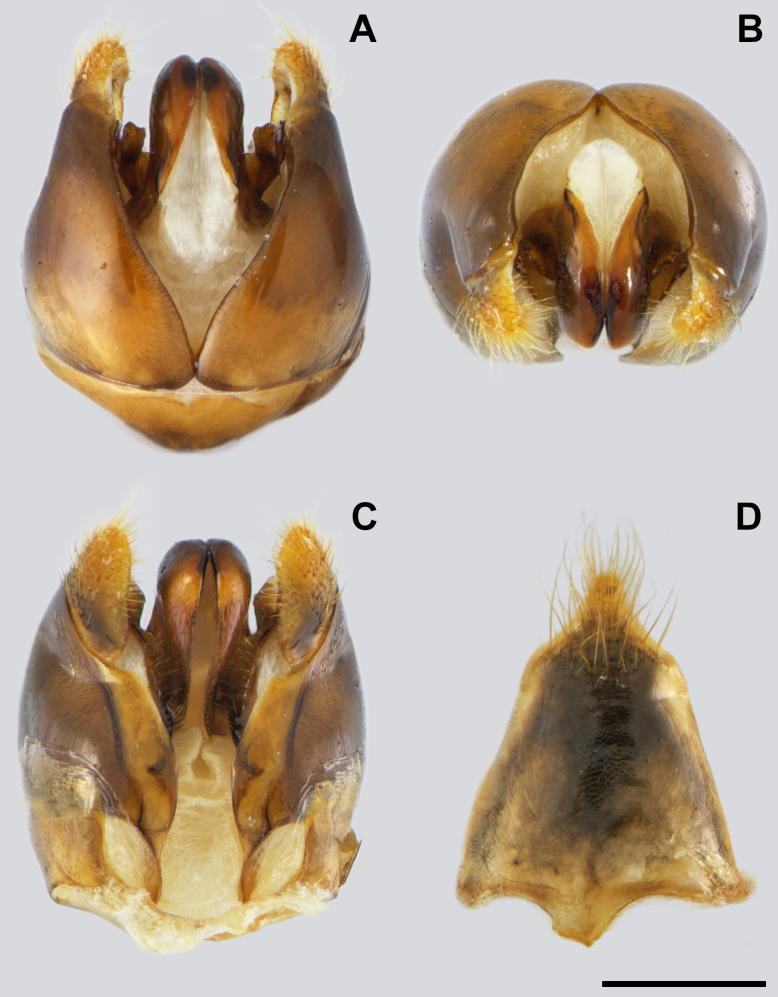
*Messor odrysarum* sp. nov., male, genitalia: **A**. dorsal view; **B**. apex; **C**. ventral view; **D**. abdominal sternite (photographed by A. Lapeva-Gjonova). Scale bar: 0.5 mm.

#### Other material.

Bulgaria • 3 w. (pin): Eastern Rhodopes, Kardzhali district, Oreshari vill., 41°36.59'N, 25°43.04'E, alt. 111 m, 22 Apr. 2014, leg. A. Lapeva-Gjonova (BFUS: BFUS-I-AG000165, BFUS-I-AG003173, BFUS-I-AG003174, BFUS-I-AG003176); • 1 g., 2 w. (pin): Eastern Rhodopes, Haskovo district, Chernichino vill., 41°35.35'N, 25°50.91'E, alt. 647 m, 23 Apr. 2021, leg. A. Lapeva-Gjonova (BFUS: from BFUS-I-AG003183 to BFUS-I-AG003185); • 10 w. (pin): same locality as previous, 14 Sept. 2025, leg. A. Lapeva-Gjonova (BFUS: from BFUS-I-AG003186 to BFUS-I-AG003195); • 7 w. (1 EtOH/6 pin): Eastern Rhodopes, Kardzhali district, Maslinovo vill., 41°46.03'N, 25°32.56'E, alt. 362 m, 03 June 2025, leg. A. Lapeva-Gjonova (BFUS: BFUS-I-AG003169; from BFUS-I-AG003177 to BFUS-I-AG003182).

#### Etymology.

The name refers to the ancient Thracian state of Odrysia, whose geographical extent included the area where the new species was found. The name is formed as a first-declension plural noun.

#### Diagnosis.

*Messor odrysarum* sp. nov. is assigned to the *Messor structor* group based on well-developed head and mesosoma sculpture, dense pilosity with erect mesosoma setae, absence of a true psammophore, and confirmed by COI analysis. The new species is most closely related to the Balkan-Anatolian species *M. oertzeni*, as indicated by the following characteristics: the longer antennal scape (SL/HW of 0.89 ± 0.05 in major workers and 1.09 ± 0.02 in gynes), the straight posterior head margin, the distinctly angulate propodeum in major workers, and the COI genetic distance of 5.72%, which is the smallest compared to other species in the genus from the region. Workers of *Messor odrysarum* sp. nov. differs from *M. oertzeni*, in their smaller body size, uniform black colouration, narrowed head posterior to the eyes, more irregular sculpture on the head and promesonotum, and longer, denser setae on the head and mesosoma, but sparser on the metasoma. The gyne of *M. odrysarum* sp. nov. can be distinguished from *M. oertzeni* by its smaller size, uniformly black body colour and less regular sculpture on the occipital part of the head and dorsal mesosoma. The new species is currently known from the East Rhodopes in Bulgaria, while *M. oertzeni* is more widely distributed across the eastern Balkans and Anatolia.

#### Description.

***Major workers* (*N* = 20)**. Measurements: HL 1.99 ± 0.15 (1.73–2.24); HW 2.01 ± 0.19 (1.64–2.31); HL/HW 0.99 ± 0.03 (0.92–1.06); SL 1.78 ± 0.10 (1.62–1.93); SL/HW 0.89 ± 0.05 (0.80–0.99); EL 0.38 ± 0.04 (0.32–0.45); MW 1.30 ± 0.11 (1.09–1.48); PSL 0.30 ± 0.04 (0.25–0.38); PTL 0.70 ± 0.06 (0.57–0.82); PTH 0.55 ± 0.04 (0.48–0.62); PTH/PTL 0.79 ± 0.05 (0.70–0.86); WL 2.62 ± 0.19 (2.37–2.95); HFL 2.45 ± 0.15 (2.18–2.66); HFL/WL 0.93 ± 0.02 (0.90–0.99).

***Colour***. Entire body shiny black, except for lower part of the genae, mandibles, antennal condyle, tip of last antennal segment, and joints of legs and tarsi, which are reddish.

***Head***. Almost squared, HL/HW 0.99, sides slightly converging towards posterior margin, latter straight. Anterior clypeal margin slightly convex, finely crenulate, without median emargination, with a row of 8 long marginal, yellowish setae. Clypeus without appressed pubescence, with few short, erect setae. Central plate of clypeus and clypeal alae with longitudinal rugae without median keel, interspaces smooth, shiny. Eyes small and broadly oval (mean EL 0.378 mm). Frontal triangle shallowly impressed, with several longitudinal rugae and smooth interspaces, shiny. Frontal carinae extend beyond frontal lobes and merge with one of antennal semicircular rugae. Frons narrow, approximately 0.25 width of head at narrowest point. Antennal fossa deep and completely surrounded by semicircular rugae, its surface smooth and shiny. Head with longitudinal striation on genae and frons and with more irregular sculpture, being mixture of microreticulate and rugulose structures on vertex. Narrow median band from vertex to frons smooth. Anterior surface of head covered with short, sparse, yellowish appressed setae, while long, decumbent to semierect setae cover its entire surface. Ventral part of head and inner margin of mandibles with numerous, semierect setae of various lengths, straight to occasionally C-shaped, which do not form distinct psammophore. Antennae elongated, 0.80–0.99 as long as head width. At base, scape strongly extended, forming rounded outer and subangulate inner angles. In frontal view, apical part of scape slightly curved with preapical constriction. Funiculus distinctly longer than scape, pedicel moderately elongated, approximately 2.2 × as long as wide at apex, flattened dorsoventrally, approximately 0.87 × as long as segments 2 and 3 combined and 1.75 × as long as segment 2. Scape with diffused microreticulation, shiny, covered with long, dense, yellowish setae, decumbent to subdecumbent. Mandibles rounded, shiny surface with deep grooves with a few yellow setae, cutting edge in large majors without teeth or with serrulate edge.

***Mesosoma***. Moderately long, approximately twice as long as wide. Promesonotum convex in lateral view with mesonotum slightly bulging above pronotal plate. Pronotum rounded at sides. Propodeum lower than promesonotum, flat dorsally and distinctly angulate; in largest workers with subangulate denticle; posterior slope oblique. Pronotum anteriorly and dorsally with distinct irregular reticulate sculpture, laterally with longitudinal rugae, interspaces smooth and shiny. Elevated dorsal plate of mesonotum with transverse rugae; sides reticulate; mesopleura with sharp, sparsely arranged, vertically positioned rugae; interspaces microreticulate and shiny. Propodeum dorsally and on posterior slope with sharp transverse rugae; metapleural area with sharp longitudinal rugae; interspaces with diffuse microreticulation; metapleurum and posterior face of propodeum partly smooth and shiny. Promesonotum, dorsal part of propodeum and mesometapleural sulcus with short, sparse, yellowish appressed setae. Numerous long, dense, erect setae cover mesosoma dorsally, longer on promesonotum and twice shorter on propodeum.

***Waist and gaster***. Moderately long peduncle with moderately high triangular node, thin, PTH/PTL 0.79. Peduncle, and base of node with distinct reticulate sculpture, anterior face concave with distinct microreticulation, slightly dull, sides and posterior face of node strongly microreticulate with several irregular rugosities. Dorsal petiole angulate in lateral view, upper margin shallowly emarginate, top and lateral sides of node with 10–15 erect setae. Postpetiole rounded in lateral view, globular in dorsal view, 1.1–1.3 × as wide as the petiole; surface microreticulate; sides and posterior face with several sharp, irregular to transverse rugosities. Dorsal sclerite with c. 20 long erect setae; sternal sclerite with c. 10 shorter erect setae. First gastral tergite entirely microreticulate; surface shiny, bearing extremely sparse and short appressed setae together with long, semierect setae, the longest reaching 0.29 in length.

***Legs***. Elongate, femora moderately swollen centrally, tibiae moderately widened apically, mid and hind tarsi longer than tibiae. Whole femora microreticulate, dorsally and laterally covered with dense and long, decumbent and subdecumbent and ventrally semierect to erect setae. Tibiae covered with dense and long decumbent to semierect setae.

***Smallest workers* (*N* = 15)**. Measurements: HL 1.01 ± 0.07 (0.89–1.15); HW 0.95 ± 0.07 (0.83–1.08); HL/HW 1.07 ± 0.02 (1.04–1.13); SL 1.03 ± 0.08 (0.92–1.17); SL/HW 1.09 ± 0.02 (1.04–1.12); EL 0.20 ± 0.02 (0.17–0.25); MW 0.64 ± 0.04 (0.57–0.70); PTL 0.39 ± 0.03 (0.31–0.43); PTH 0.29 ± 0.02 (0.27–0.33); PTH/PTL 0.76 ± 0.04 (0.71–0.85); WL 1.40 ± 0.09 (1.23–1.53); HFL 1.20 ± 0.10 (1.02–1.37); HFL/WL 0.86 ± 0.05 (0.68–0.91).

***Colour***. Same as major workers, black, the antennae, legs, waist and gaster appear dark brown.

***Head***. Slightly more elongated (HL/HW mean 1.07) and more rounded in frontal view than major workers, softly converges posterad behind eyes, posterior head corners rounded, and occipital margin straight. Antennae elongated; scape 1.04–1.12 as long as head width, covered with long, dense erect setae. Sculpture of head less developed than in major workers, with striation on frontal area and genae, vertex and occipital corners with microreticulation only.

***Mesosoma***. As slim as majors, ML/MW ratio mean 2.2. Sculpture of mesosoma as in major workers, lateral sides of pronotum striate with microreticulation between striae. Propodeum rounded in lateral view, not angled and without denticle. Setation of mesosoma as in majors but with lower number of setae.

***Waist and gaster***. Waist as in major workers, but with smaller number of erect setae (fewer than 10) and with microreticulation only, without rugosity. Gaster microreticulate and shiny. Rest of characters as in major workers.

***Legs***. Elongate; femora moderately swollen medially, tibiae moderately widened preapically; mid and hind tarsi longer than tibiae. Femora entirely microreticulate, dorsally and laterally with short decumbent to subdecumbent setae, ventrally with longer decumbent to semierect setae. Tibiae with dense decumbent to semierect setae.

**Gynes (*N* = 3). *Measurements***: HL 2.18 ± 0.05 (2.12–2.22); HW 2.19 ± 0.07 (2.11–2.24); HL/HW 0.99 ± 0.05 (0.95–1.05); SL 1.85 ± 0.03 (1.82–1.88); SL/HW 0.84 ± 0.04 (0.82–0.89); EL 0.55 ± 0.00 (0.55–0.56); MW 2.08 ± 0.04 (2.03–2.11); PSL 0.56 ± 0.03 (0.53–0.60); PTL 1.26 ± 0.09 (1.17–1.35); PTH 0.91 ± 0.02 (0.90–0.93); PTH/PTL 0.73 ± 0.04 (0.69–0.77); WL 3.93 ± 0.12 (3.82–4.07); HFL 2.67 ± 0.02 (2.66–2.69); HFL/WL 0.68 ± 0.02 (0.66–0.70).

***Colour***. Entire body shiny black, except for lower part of the genae, mandibles, antennal condyle, last four antennal segments, joints of legs and tarsi, reddish.

***Head***. Almost squared, HL/HW 0.99, sides subparallel, the posterior head corners rounded and posterior margin straight. Anterior clypeal margin convex, finely crenulate, without median emargination, with row of long marginal, yellowish setae. Clypeus with appressed pubescence. Central plate of clypeus and clypeal alae with longitudinal rugae without median keel, interspaces smooth, shiny. Eyes small and broadly oval (mean EL 0.55 mm). Frontal triangle shallowly impressed, with several longitudinal rugae and smooth interspaces, shiny. Frontal carinae short, slightly extending beyond frontal lobes. Frons narrow, at narrowest point approximately 0.3 width of head. Antennal fossa deep and completely surrounded by semicircular rugae, its surface smooth and shiny. Head with longitudinal striation on genae and frons and with more irregular rugulose sculpture on vertex. Anterior surface of head covered with short, sparse, yellowish appressed setae, while subdecumbent to decumbent setae cover entire surface. Ventral part of head and inner margin of mandibles with numerous, semierect setae of various lengths, straight to occasionally C-shaped, which do not form a distinct psammophore. Antennae elongated, 0.82–0.89 as long as head width. At its base, scape strongly extended, forming rounded outer and subangulate inner angles. In a frontal view, apical part of scape slightly curved with preapical constriction. Funiculus distinctly longer than scape, pedicel moderately elongated, approximately twice as long as wide at apex, approximately 0.83 × as long as segments 2 and 3 combined and 1.58 × as long as segment 2. Scape with diffused microreticulation, shiny, covered with long, dense, yellowish setae, appressed to decumbent.

Mandibles rounded, surface shiny with deep grooves with dense yellow setae, cutting edge with large, sharp teeth.

***Mesosoma***. Stout and moderately long, approximately twice as long as wide (WL/MW 1.9). Pronotum not visible from above and rounded at sides. Its anterior slope scabrous, while laterally it is covered with longitudinal rugae, interspaces smooth and shiny. Scutum slightly convex above pronotal plate. Its surface smooth and shiny along the midline, and punctate on sides. Posterior half with irregular longitudinal rugosity. Scutellum slightly convex, almost smooth, shiny. Anepisternum, katepisternum and lateral sides of propodeum with longitudinal rugae; interspaces smooth and shiny. Metanotum and posterior slope of propodeum with distinct, regular, transverse rugae. Propodeal spines moderately long, in form of triangular teeth, PSL 0.56 ± 0.03. Dorsal surface of scutum covered with dense, long, yellowish setae, longest of 0.461 mm, sparser on scutellum, and shortest on dorsal side of propodeum.

***Waist and gaster***. Petiole elongate, with long peduncle and moderately high triangular node, thin, PTH/PTL 0.73, peduncle, and anterior face of node with microreticulation, and posterior face with sharp transverse rugae. Upper margin of petiole straight or slightly emarginate, top and posterior face with erect yellow setae. Postpetiole rounded in lateral view, globular in dorsal view, 0.75 × as wide as petiole, anterior face smooth and shiny, on sides and on posterior face with perpendicular and transverse rugae, with long erect setae. First gastral tergite entirely microreticulate; surface shiny, covered with short appressed setae together with long, subdecumbent to semierect setae, the longest reaching 0.4 in length.

***Legs***. Legs elongate; femora slightly swollen medially, tibiae moderately widened preapically, mid and hind tarsi longer than tibiae. Femora microreticulate, dorsally with dense decumbent setae, laterally with decumbent to subdecumbent, ventrally with subdecumbent to semierect setae. Tibiae with dense, long, decumbent to semierect setae.

**Males (*N* = 3). *Measurements***: HL 1.24 ± 0.03 (1.21–1.26); HW 1.28 ± 0.05 (1.22–1.30); HL/HW 0.97 ± 0.05 (0.93–1.02); SL 0.50 ± 0.07 (0.44–0.58); SL/HW 0.39 ± 0.07 (0.34–0.48); EL 0.50 ± 0.01 (0.48–0.51); MW 1.65 ± 0.05 (1.60–1.70); PSL 0.38 ± 0.02 (0.36–0.39); PTL 0.94 ± 0.08 (0.85–0.99); PTH 0.52 ± 0.0 (0.52–0.53); PTH/PTL 0.56 ± 0.05 (0.53–0.61); WL 3.22 ± 0.06 (3.15–3.26); HFL 2.81 ± 0.05 (2.76–2.86); HFL/WL 0.87 ± 0.01 (0.87–0.88).

***Colour***. Body shiny black; incisor part of mandibles, tip of the last antennal segment, leg joints, and tarsi reddish.

***Head***. Head slightly wider than long (HL/HW 0.97); posterior head corners broadly rounded, posterior margin slightly convex. Mandibles triangular, smooth basally and apically, striate medially, bearing eight acute teeth, apical and preapical largest. Clypeus convex medially, without median emargination; surface with several irregular rugae, interspaces microreticulate. Frontal carinae short, not extending beyond midpoint of eyes. Lower margin of antennal socket below lower eye margin. Ocelli well below posterior head margin; anterior ocellus above level of upper eye margin. Eyes large (mean EL 0.50), strongly convex, situated closer to anterior than to posterior head margin, with microscopic hairs. Antenna 13-segmented, without apical club, last segment 1.6 × longer than the previous one. Head mainly microreticulate, with longitudinal striation on the genae and frons, and covered with long, dense, yellowish setae, longest on posterior corners and margin, and clypeal margin.

***Mesosoma***. Stout; mesoscutum enlarged and strongly convex, overhanging pronotum; parapsidal lines distinct. Mesoscutellum convex. Metanotum short, vertical, with transverse rugae. Propodeum angulate in lateral view; surface with longitudinal rugosity, interspaces microreticulate. Metasternal process large, longer than high, apex dentate. Mesosoma densely covered with long yellowish setae, erect dorsally and decumbent laterally.

***Waist and gaster***. Waist elongate, with long peduncle and triangular petiole (PTH/PTL 0.56). Peduncle, anterior face, and lateral sides of petiole microreticulate; posterior face with median longitudinal rugae and reticulate laterally. Petiole with long, curved, backward-directed setae on the upper margin and posterior face, and short, erect to appressed setae on the anterior face. Postpetiole rounded in lateral view and globular in dorsal view, 1.2 × as wide as petiole; surface reticulate with sparse low rugae. Dorsal surface with long curved backward setae and decumbent setae posteriorly; ventrally with erect setae. First gastral tergite microreticulate; surface shiny, covered with long appressed setae together with long, subdecumbent to semierect setae; midline hairless.

***Legs***. Elongate; mid and hind tarsi longer than tibiae. Femora and tibiae microreticulate; dorsally with decumbent setae, denser apically; laterally with decumbent to subdecumbent setae; ventrally with decumbent to semierect setae. Tibiae dorsally with dense, short, decumbent to subdecumbent setae.

#### COI barcode.

A sequence of the species has been deposited in BOLD (accession number BGANT009-23, BIN: BOLD:AES9246) and analysed by Lapeva-Gjonova et al. (2025) under the provisional name *Messor* sp. 2. According to the Kimura 2-parameter model, the new species shows a COI genetic distance of 5.72% from *M. oertzeni*. Phylogenetic reconstruction using both Bayesian inference and maximum likelihood indicates that it is a sister taxon of *M. oertzeni*, with very strong support (1.00 posterior probability and 95% bootstrap value, respectively) (see Lapeva-Gjonova et al. 2025 for details).

#### Biological notes.

**Figure 10.**
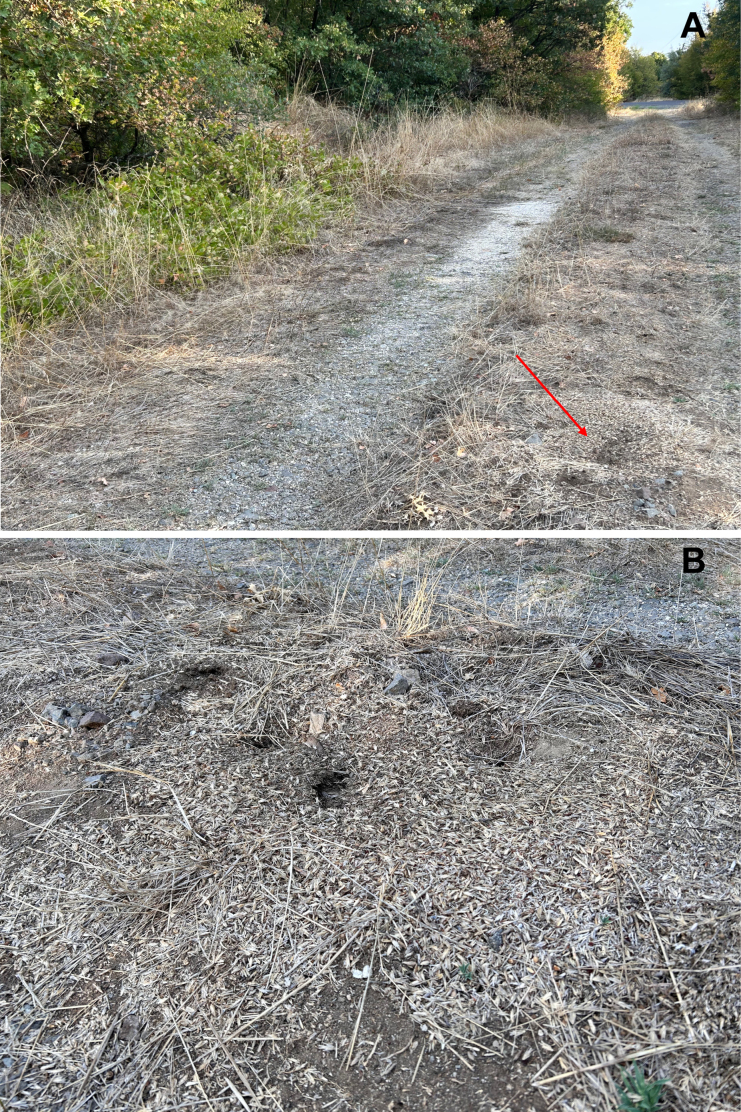
*Messor odrysarum* sp. nov.: **A**. type locality; **B**. nest construction (photographed by A. Lapeva-Gjonova).

A lowland species, occurring at elevations up to 647 m. Nests were found in the middle of dirt roads within oak forests or open grasslands, with entrances at ground level. In late summer, seed remains were observed near the nest entrances (Fig. 10). Alate gynes and males were collected intranidally in April and September. Syntopic occurrence was recorded with *Messor wasmanni* and *M. oertzeni*. The myrmecophilous beetle *Cholovocera balcanica* Karaman, 1936 (Coleoptera: Endomychidae) was found in large numbers in the nests of the new species at the type locality (Sokolyane), Oreshari and Chernichino.

#### Distribution.

*Messor odrysarum* sp. nov. is currently only known from the Eastern Rhodopes in Bulgaria, possibly also occurring in the Thracian region of Greece and Türkiye.

### Messor oertzeni

Taxon classificationAnimaliaHymenopteraFormicidae

Forel, 1910

EC6BF84D-7E8A-5D05-AC50-C5B52536088F

Figs 11, 12

#### Material examined.

Bulgaria • 1 g. (pin), Struma valley, Rupite; 41°27.12'N, 23°16.05'E, alt. 91 m, 23 Aug. 2025, leg. A. Lapeva-Gjonova, Catalog Number: BFUS-I-AG003234 (BFUS).

**Figure 11.**
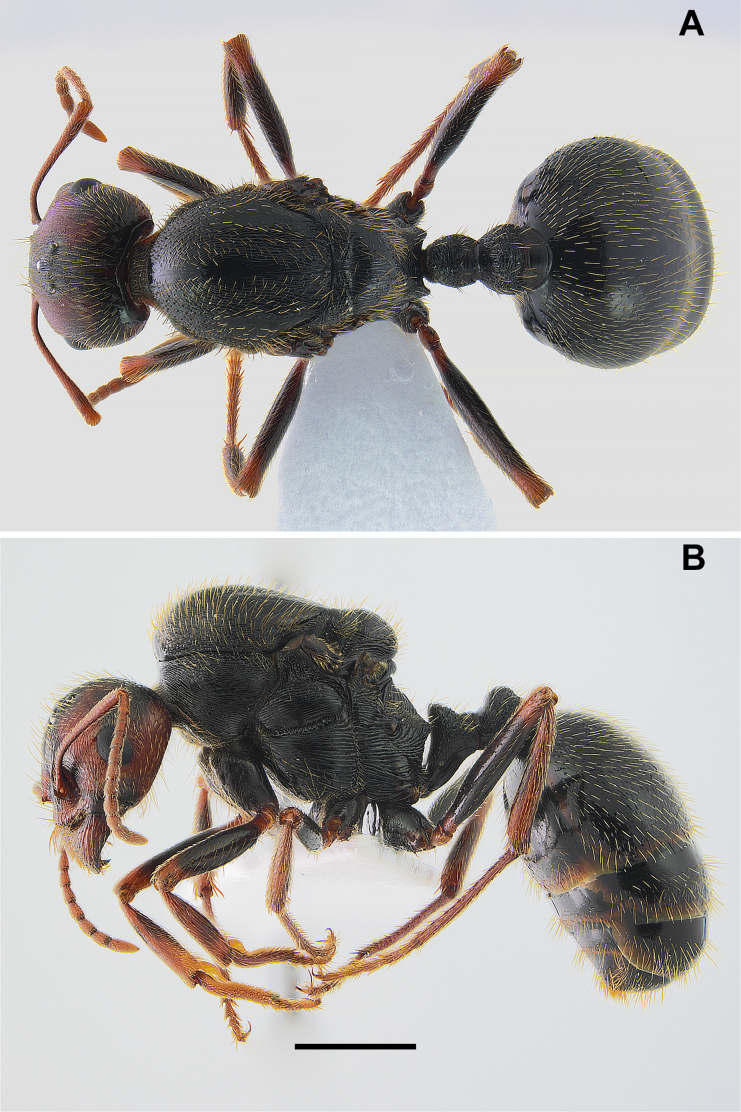
*Messor oertzeni*, gyne: **A**. dorsal view; **B**. lateral view (photographed by A. Lapeva-Gjonova). Scale bar: 2 mm.

**Figure 12.**
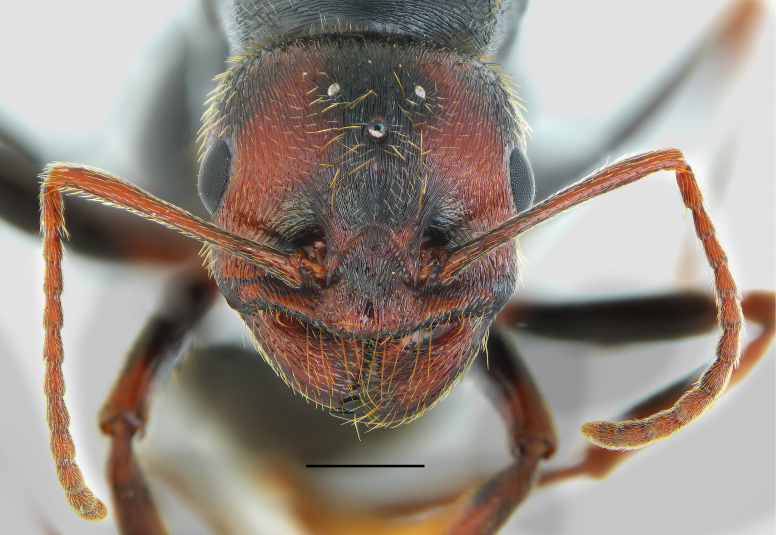
*Messor oertzeni*, gyne head (photographed by A. Lapeva-Gjonova). Scale bar: 1 mm.

#### Redescription.

**Gyne (*N* = 1). *Measurements***: HL 3.37; HW 2.50; HL/HW 1.35; SL 2.28; SL/HW 0.91; EL 0.64; MW 2.40; PSL 0.66; PTL 1.43; PTH 1.03; PTH/PTL 0.72; WL 4.56; HFL 3.09; HFL/WL 0.68.

***Colour***. Head reddish, with posterior head corners, a medial band extending from posterior head margin to central part of clypeus, antennal fossae, and clypeal margins dark brown to black. Antennae uniformly reddish. Mesosoma, waist and gaster black. Legs with coxae and median portion of femora dark brown to black; remaining parts reddish to brown.

***Head***. Rectangular, longer than wide (HL/HW 1.35); sides subparallel. Posterior corners of head rounded; posterior margin straight. Clypeus with appressed pubescence and few short, erect setae. Anterior clypeal margin slightly convex, finely crenulate, without a median emargination, bearing row of ten long, yellowish marginal setae. Central plate and clypeal alae with longitudinal rugae, lacking median keel; interspaces smooth and shiny. Eyes small, broadly oval (EL 0.643 mm). Frontal triangle shallowly impressed, with longitudinal rugae and smooth, shiny interspaces. Frontal carinae extending beyond frontal lobes and merging with one of semicircular antennal rugae. Frons approximately one-third of width of head. Antennal fossa deep, completely surrounded by semicircular rugae; surface smooth and shiny. Head with extremely dense longitudinal striation, slightly divergent around eyes and in occipital corners. Genae, lateral sides of head and occipital region with moderately dense and short decumbent to semierect setae, longest in occipital corners with length 0.205. Ventral part of head with numerous, semierect setae of various lengths, straight to occasionally C-shaped, which do not form a distinct psammophore. Antennae elongated, 0.91 as long as head width. At its base, the scape is strongly extended, forming rounded outer and subangulate inner angles. In a frontal view, the apical part of the scape is slightly curved with a preapical constriction. Funiculus distinctly longer than scape, pedicel moderately elongated, approximately 2.2 × as long as wide at apex, flattened dorsoventrally, approximately 0.75 × as long as segments 2 and 3 combined and 1.43 × as long as segment 2. Scape with microreticulation, shiny, covered with short, dense, setae, decumbent to subdecumbent. Mandibles rounded, shiny surface with deep grooves with dense yellow setae, cutting edge with large, sharp teeth.

***Mesosoma***. Stout and moderately long, approximately 2 × as long as wide (WL/MW 1.9). Pronotum is not visible from above and is rounded at the sides. Its anterior slope with transverse rugae, lateral sides covered with longitudinal rugae, interspaces smooth and shiny. Scutum slightly convex above pronotal plate. Its surface smooth and shiny on most convex central and lateral areas and longitudinally striate in areas between them. Scutellum slightly convex, longitudinally striate, shiny. Anepisternum, katepisternum and lateral sides of propodeum with longitudinal rugae; interspaces smooth and shiny. Metanotum and posterior slope of propodeum with distinct, regular, transverse rugae. Propodeal spines moderately long, in form of triangular teeth, PSL 0.66. Dorsal surface of scutum covered in dense, long, yellowish setae, the longest of 0.401 mm; sparser on scutellum, and shortest on dorsal side of propodeum.

***Waist and gaster***. Petiole elongate, with long peduncle and moderately high triangular node, thin, PTH/PTL 0.72, peduncle, and anterior face of node with microreticulation, and posterior face on the top with sharp transverse rugae. Upper margin of petiole slightly emarginate, top and posterior face with erect yellow setae. Postpetiole rounded in lateral view, globular in dorsal view, 0.76 × as wide as petiole, anterior face finely striate, lateral and posterior faces reticulate, with long, erect, backward-directed setae. First gastral tergite entirely microreticulate; surface shiny, covered with short appressed setae together with moderately long, subdecumbent to semierect setae, longest reaching 0.294 in length.

***Legs***. Elongate; femora slightly swollen medially, tibiae moderately widened preapically, mid and hind tarsi longer than tibiae. Femora microreticulate, dorsally with decumbent setae, laterally with decumbent to subdecumbent, ventrally with subdecumbent to semierect setae. Tibiae with dense, decumbent to subdecumbent setae.

##### Key to *Messor* species in Bulgaria

**Table:** 

1	First metasomal segment on dorsal side without or with single erect setae	**2**
–	First metasomal segment on dorsal side with numerous erect setae	**3**
2	Larger in size, average head width of major workers is over 2.6 mm. Mesosoma of major workers usually completely red or with red-brown spots, but never completely black. Occipital area and vertex usually with less than 12 erect setae	***M. wasmanni***
–	Smaller in size, average head width of major workers is less than 2.5 mm. Mesosoma of major workers is usually black. Occipital area and vertex usually with 12–20 erect setae	***M. atanassovii***
3	Head ventrally with strongly developed psamophore (long, J-shaped setae). Sculpture of the head smooth or with slight longitudinal striations in major workers	***M. hellenius***
–	Head ventrally with short and straight setae. Sculpture of the head is with well-developed longitudinal striation in major workers	**4**
4	Antennal scapes longer, with an SL/HW ratio 0.82–0.91 in major workers	**5**
–	Antennal scapes shorter, with an SL/HW ratio 0.61–0.78 in major worker	**6**
5	Larger species, WL in largest major workers 3.75–4.05, HW 2.73–2.89. Usually head and mesosoma uniformly reddish, contrasting with the brownish petiole, postpetiole and metasoma, occasionally head and mesosoma with brown patches of diffused borders	***M. oertzeni***
–	Smaller species, WL in largest major workers 2.37–2.95, HW 1.64–2.31. Body uniformly black	***M. odrysarum* sp. nov**.
6	Head with a reduced number of erect setae on the lateral margins; finely sculptured surface of the head, the postocular area often mostly smooth	***M. ponticus***
–	Head with numerous erect setae on lateral margins; strongly sculptured surface of head, postocular area often completely or mostly striate	**7**
7	First tergite of metasoma with highly developed imbricate microsculpture. First segment of antennal flagellum not flattened and shorter than next two segments combined	**8**
–	First tergite of metasoma smooth, with weak imbricate microsculpture. First segment of antennal flagellum strongly elongated and flattened, almost as long as next two segments combined	***M. structor***
8	Head with very regular striated sculpture and body as a whole, including the petiole. Posterior margin of head in major workers deeply concave. Large species, WL of major workers 3.20–3.48, HW 2.93–3.25, WL of gynes 4.53–4.90, HW 2.20–2.43	***М. mcarthuri***
–	Head with less regularly striated sculpture. Posterior margin of head in major workers very shallowly concave or straight. Small species, WL of major workers 2.70–3.00, HW 2.37–2.60, WL of gynes 3.07–3.20, HW 1.74–1.80	***M. ibericus***

## Supplementary Material

XML Treatment for Messor odrysarum

XML Treatment for Messor oertzeni

## Additional information

### Use of AI

No use of AI was reported.

### Author ORCIDs

Albena Lapeva-Gjonova https://orcid.org/0000-0003-0811-0768

Lech Borowiec https://orcid.org/0000-0001-5668-6855

### Data availability

All of the data that support the findings of this study are available in the main text.
